# Progressive search in tandem mass spectrometry

**DOI:** 10.1186/s12859-023-05222-2

**Published:** 2023-03-14

**Authors:** Yoonsung Joh, Kangbae Lee, Hyunwoo Kim, Heejin Park

**Affiliations:** 1 Department of Computer Science, Hanyang University, Seoul, 06978 Republic of Korea; 2 Biomedical Informatics Team, Korea Institute of Science and Technology Information, Daejeon, 34141 Republic of Korea

**Keywords:** Mass spectrometry, Protein sequence analysis, Proteomics, Algorithms

## Abstract

**Background:**

High-throughput Proteomics has been accelerated by (tandem) mass spectrometry. However, the slow speed of mass spectra analysis prevents the analysis results from being up-to-date. Tandem mass spectrometry database search requires *O*(|*S||D|*) time where *S* is the set of spectra and *D* is the set of peptides in a database. With usual values of |*S|* and |*D|*, database search is quite time consuming. Meanwhile, the database for search is usually updated every month, with 0.5–2% changes. Although the change in the database is usually very small, it may cause extensive changes in the overall analysis results because individual PSM scores such as deltaCn and E-value depend on the entire search results. Therefore, to keep the search results up-to-date, one needs to perform database search from scratch every time the database is updated, which is very inefficient.

**Results:**

Thus, we present a very efficient method to keep the search results up-to-date where the results are the same as those achieved by the normal search from scratch. This method, called progressive search, runs in *O*(|*S||ΔD|*) time on average where *ΔD* is the difference between the old and the new databases. The experimental results show that the progressive search is up to 53.9 times faster for PSM update only and up to 16.5 times faster for both PSM and E-value update.

**Conclusions:**

Progressive search is a novel approach to efficiently obtain analysis results for updated database in tandem mass spectrometry. Compared to performing a normal search from scratch, progressive search achieves the same results much faster. Progressive search is freely available at: https://isa.hanyang.ac.kr/ProgSearch.html.

## Background

Database search in tandem mass spectrometry, usually done by as Sequest [1], Tide [2], Comet [3], Mascot [4], Maxquant [5], MS-GF [6], MSFragger [7], and so on, is quite time consuming: Especially when the number of spectra is large, for example, more than 10 million of spectra [8, 9] and/or the search space is wide such as open search [7].

Meanwhile, protein databases used for search are updated frequently. For example, the most widely used database, Uniprot [10], is updated monthly with 0.5 to 2% changes, which means newly identified protein sequences are inserted and some incorrect sequences are deleted. Although the change is very small, it may cause changes in the overall analysis results because each spectrum score is calculated relatively based on the entire search results. Therefore, to keep the search results up-to-date, one needs to perform database search from scratch every time the database is updated, which is very inefficient.

**Fig. 1:**
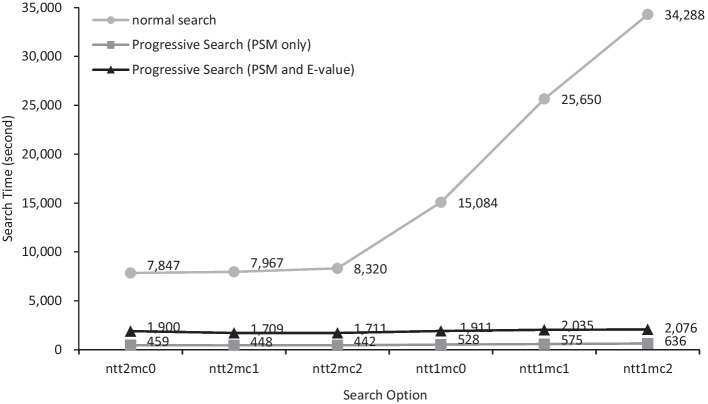
Search time comparison between the normal search from scratch and the progressive search

**Fig. 2:**
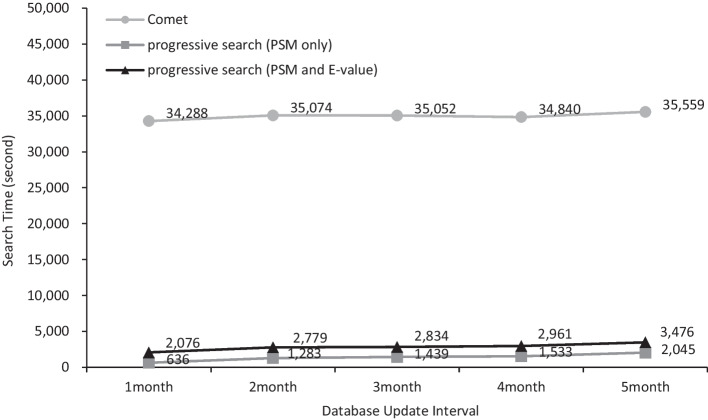
Search time comparison according to database update intervals for ntt1mc2

Thus, we present a very efficient method to keep the search results up-to-date where the results are the same as those achieved by the normal search from scratch. This method, called progressive search, efficiently minimizes the computation time such that our progressive search is much faster than the normal search from scratch. In this study, we applied our progressive search to Comet which is not only incorporated into widely used proteomics pipelines such as Trans-Proteomics Pipeline [11] and Crux [12] but also a stand-alone open source tandem mass spectrometry database search engine. Our experimental results in Figs. 1 and 2 show that progressive search is 16.5–53.9 times faster than the normal search where the database change is 0.16%, the number of tryptic termini is 1, and the number of missed cleavage is 2.

## Implementation

### Database separation

**Fig. 3:**
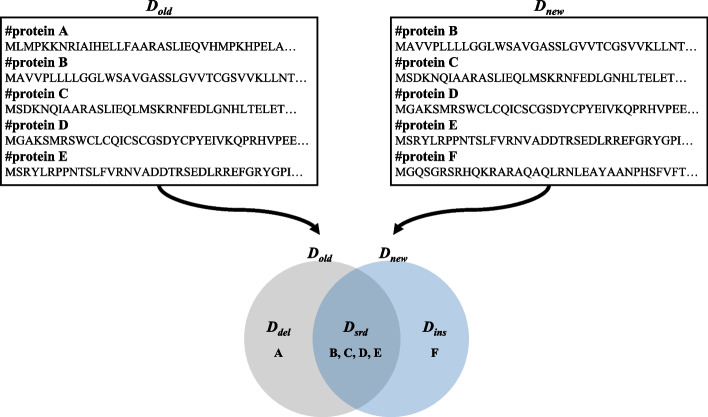
Database comparison example

First, we compare the old database *D*_*old*_ and the new database *D*_*new*_ to identify *D*_*srd*_, *D*_*del*_, and *D*_*ins*_ where *D*_*srd*_ contains the proteins shared by both *D*_*old*_ and *D*_*new*_, *D*_*del*_ contains the proteins stored in only *D*_*old*_, and *D*_*ins*_ contains the proteins stored in only *D*_*new*_. Let *R*_*old*_, *R*_*new*_, and *R*_*srd*_ denote the PSM results for *D*_*old*_, *D*_*new*_, and *D*_*srd*_, respectively. Figure 3 shows the case that *D*_*old*_ is the set of proteins {A, B, C, D, E} and *D*_*new*_ is the set of proteins {B, C, D, E, F}. Thus, *D*_*srd*_ is the set of proteins {B, C, D, E}, *D*_*del*_ is {A}, and *D*_*ins*_ is {F}.

**Fig. 4:**
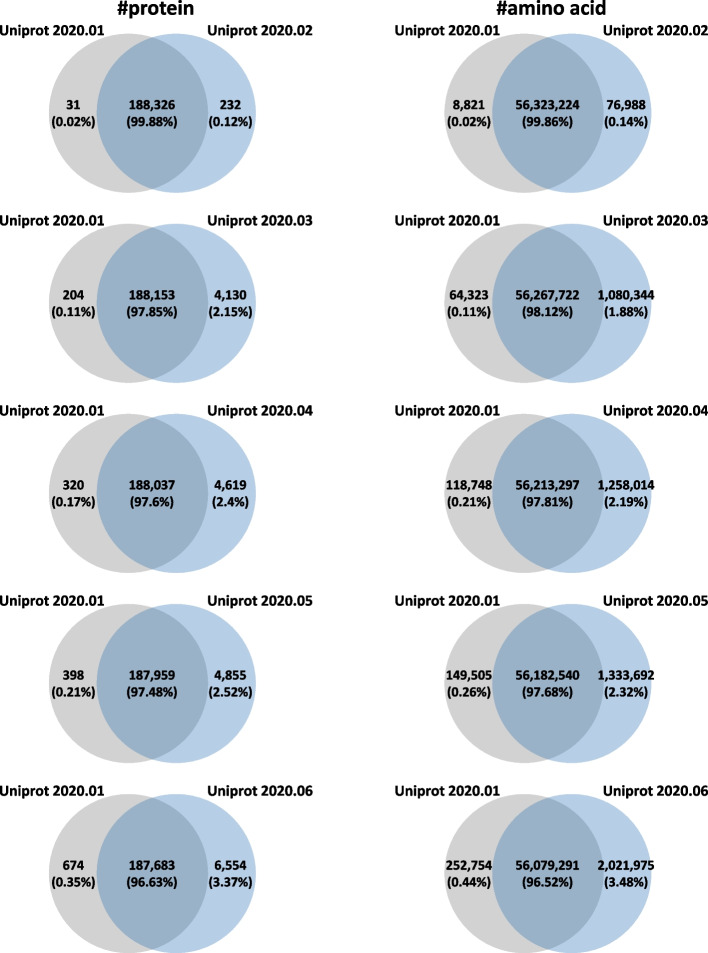
Differences between various Uniprot versions. We used different Uniprot database versions from January to June 2020 for database comparison. The databases were compared based on the number of proteins and amino acids

For experimental results, we used UniProtKB database released from January to June 2020 (Fig. 4). On average, 0.07% and 0.67% of the proteins were deleted and inserted every month, respectively. In addition, 0.09% and 0.70% of the amino acids are deleted and inserted every month on average, respectively.

### Workflow

**Fig. 5:**
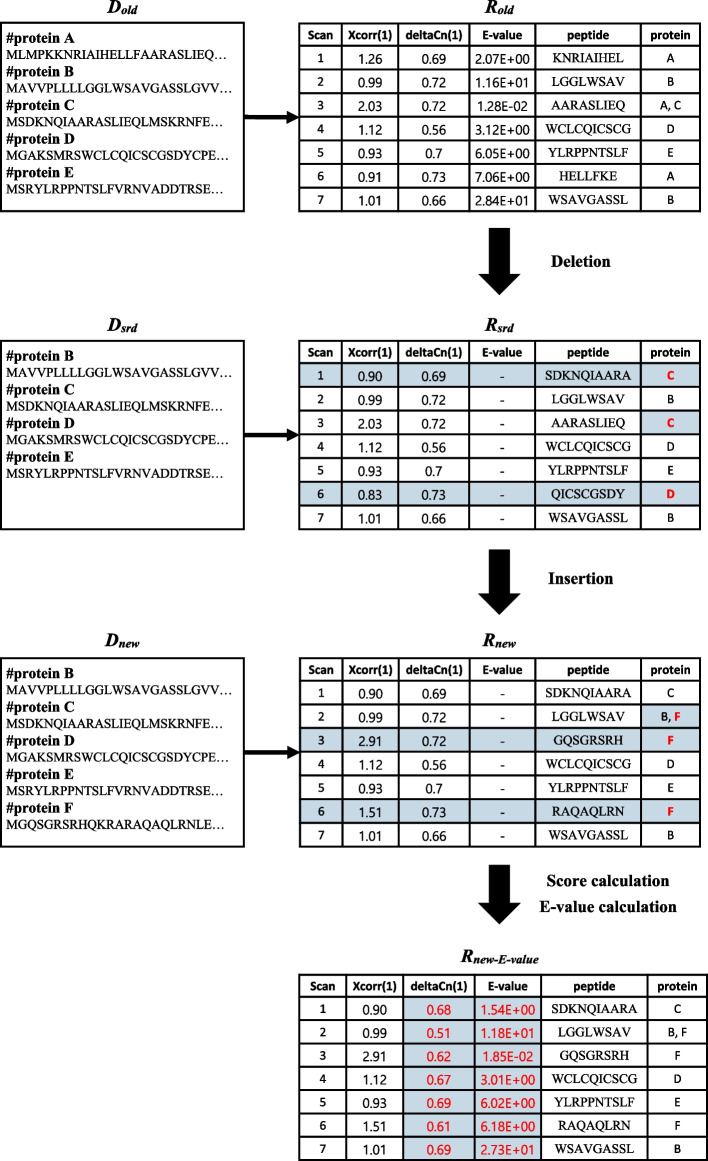
Workflow overall

Progressive search consists of four steps called “deletion”, “insertion”, “score calculation”, and “E-value calculation” (Fig. 5). We explain the progressive search that runs in *O*(|*S|*|*ΔD|*) time on average where* S* is the set of spectra and *ΔD* is the difference between the old and the new databases where |*X*| denotes the number elements in *X*.

1. *Deletion* This is the process of obtaining *R*_*srd*_ from *R*_*old*_. The *R*_*srd*_ is the same as *R*_*old*_ except the PSMs whose peptide sequences are from only *D*_*del*_*.* Those PSMs are deleted and replaced by PSMs obtained by searching *D*_*srd*_ for the spectra in the deleted PSMs (*S*_*del*_)*.* For example, PSMs of scans 1, 3 and 6 are updated after deletion in Fig. 5.
2. *Insertion* This is the process of obtaining *R*_*new*_ from *R*_*srd*_. We search *D*_*ins*_ for all the spectra to find PSMs. Then the found PSMs are compared with the PSMs in *R*_*srd*_. The PSMs with better scores are selected and stored in *R*_*new*_. For example, PSMs of scans 2, 3 and 6 are updated after insertion in Fig. 5.
3. *Score calculation* This is the process of calculating deltaCn values in *R*_*new*_. deltaCn is a score representing the difference between Xcorr values. Since we got the Xcorr of *R*_*new*_ through previous steps, we can calculate the deltaCn of *R*_*new*_ in this step.
4. *E-value calculation* This is the process of calculating E-values in *R*_*new*_. Note that the E-value of every PSM may be invalid even if only one of all PSMs has been changed. Since E-value calculation requires all PSM information that has not been output by the original Comet, we built “Comet-E”, a modified version of Comet, to address the E-value correction.

Detailed explanations are given in the following subsections: Deletion, Insertion, Score calculation, and E-value calculation.

### Deletion

**Fig. 6:**
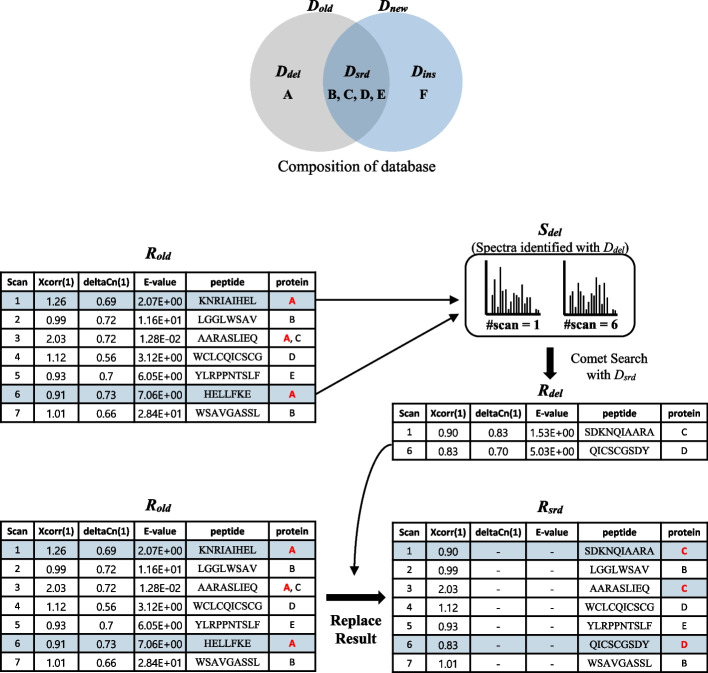
Deletion example

Algorithm description: The main purpose of deletion is converting *R*_*old*_ into *R*_*srd*_. Each spectrum in *R*_*old*_ was identified by either *D*_*del*_ or *D*_*srd*_ (Fig. 6, Composition of database). Recall that *S*_*del*_ denote the set of spectra identified by only *D*_*del*_ and let *S*_*srd*_ denote the set of spectra identified by *D*_*srd*_. While the PSMs for *S*_*srd*_ remain as they are, the PSMs for *S*_*del*_ should be replaced by the PSMs obtained by searching *D*_*srd*_ for *S*_*del*_. In the example in Fig. 6, among the PSMs for scans 1–7, only the PSMs for scans 1 and 6 are identified with only *D*_*del*_ (= {A}). Thus, *S*_*del*_ consists of spectra in scans 1 and 6. (Note that the PSM for scan 3 belongs to *S*_*srd*_ because its peptide AARASLIEQ exists in both proteins A and C and thus all we have to do is to delete A from the protein list of scan 3.) We search *D*_*srd*_ (= {B, C, D, E}) for *S*_*del*_ and the new results replace the old results of *S*_*del*_.

Time complexity: Since only *D*_*srd*_ is searched for *S*_*del*_, the time complexity is *O(|S*_*del*_*|*∙|*D*_*srd*_|). We show that *O(|S*_*del*_*|*∙|*D*_*srd*_|) is reduced to *O*(|*S*|∙|*D*_*del*_*|*) on average where *S* is the set of total spectra *S*_*del*_ ∪ *S*_*srd*_. If we assume that PSMs were randomly selected from *D*_*old*_ in general (this assumption is verified in Results), the ratio |*S*_*del*_|/|*S*| is similar to the ratio |*D*_*del*_|/|*D*_*old*_|. Thus, |*S*_*del*_*|* is approximately the same as |*S*|∙|*D*_*del*_*|/*|*D*_*old*_*|* and the time complexity can be expressed as *O*(|*S*|∙|*D*_*del*_*|*∙|*D*_*srd*_|*/*|*D*_*old*_*|*). Furthermore, since |*D*_*srd*_|*/*|*D*_*old*_*|*≤ 1, the time complexity is reduced to *O*(|*S*|∙|*D*_*del*_*|*) on average.

### Insertion

**Fig. 7:**
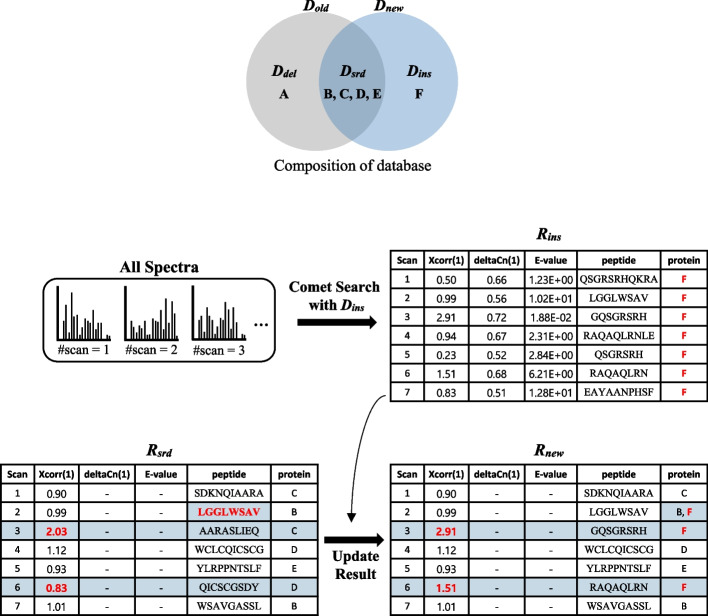
Insertion example

The main purpose of insertion is converting *R*_*srd*_ into *R*_*new*_. Each spectrum in *R*_*new*_ is identified by either *D*_*ins*_ or *D*_*srd*_. First, we search *D*_*ins*_ for the set *S*. Let *R*_*ins*_ denote the search result. For each spectrum, we replace its PSM in *R*_*srd*_ by its PSM in *R*_*ins*_ if the Xcorr of the PSM in *R*_*ins*_ is higher than that in *R*_*srd*_. In Fig. 7, we search *D*_*ins*_ (= {F}) for all the spectra and get *R*_*ins*_. Since only the PSMs of scans 3 and 6 in *R*_*ins*_ have higher Xcorr values (2.91 and 1.51) than those in *R*_*srd*_ (2.03 and 0.83), *R*_*new*_ is obtained by replacing the PSMs of scans 3 and 6 in *R*_*srd*_ with those in *R*_*ins*_. (Note that the scan 2 result of *R*_*ins*_ is the same as *R*_*srd*_ because its peptide LGGLWSAV exists in both proteins B and F and thus all we have to do is to add F to the protein list of scan 2.) Since the main part of insertion is to search *D*_*ins*_ for the set *S*, the time complexity is *O(|S|*∙|*D*_*ins*_|).

### Score calculation

After the deletion and insertion, all PSMs with their Xcorr scores have been updated for *D*_*new.*_ Now, the deltaCn values which are defined as follows should be recalculated.

$${\text{deltaCn}}\left( i \right) \, = { 1 }{-}{\text{ Xcorr}}\left( {i + { 1}} \right) \, /{\text{ Xcorr}}\left( i \right)$$

where Xcorr(*i*) denote the *i*-th largest PSM score for a spectrum. Thus, recalculating deltaCn takes *O*(|*S*|) (= *O*(|PSM|)) time in the worst case. In addition, when deltaCn is updated, there are two subtleties to consider as follows.

(i) **Increment of the parameter *num_output_lines* by 1**

In order to calculate deltaCn(*i*), not only Xcorr(*i*) but also Xcorr(*i* + 1) is required. Since the parameter *num_output_lines* of Comet determines the number of Xcorr values in the output, *num_output_lines* should be *n* + 1 if deltaCn(*i*)’s for $i\le n$ are to be calculated by progressive search (Comet-P or Comet-E). Even though Comet just outputs *n* lines, it always calculates the Xcorr values for PSMs of all ranks, and thus incrementing *num_output_lines* by 1 rarely affects the total running time.

(ii) **Xcorr precision refinement in the output**

In Comet, the internal data type of Xcorr is double but the Xcorr values in the output of Comet are rounded to the fourth decimal place as shown in Table 1. Thus, the deltaCn(1) calculated by Comet is different from the deltaCn(1) calculated by Xcorr(1) and Xcorr(2) values from the output of Comet as explained in the legend of Table 1. Hence, the Xcorr values in the output of Comet-P/Comet-E are rounded to the seventh decimal place so that the deltaCn calculated by the output of Comet-P/Comet-E is the same as that calculated by Comet.

**Table 1:** Xcorr precision refinement

	Comet (internal)	Comet (output)	Comet-P/Comet-E (output)
Xcorr(1)	0.0171654	0.0172	0.0171654
Xcorr(2)	0.0324375	0.0324	0.0324375
deltaCn(1)	0.4708162	0.4708	0.4708

Note that 0.4708, deltaCn(1) in the Comet (output) column, is achieved by rounding 0.4708162 (= 1 − 0.0171654/0.0324374) which is the deltanCn(1) in the Comet (internal) column. Thus, if deltaCn(1) is calculated from 0.0172 to 0.0324 which are the Xcorr values in the Comet (output) column, it becomes 0.4691 (= 1 −  0.0172/0.0324) which is different from 0.4708. Hence, the output of Xcorr values of Comet-P/Comet-E is rounded to the 7th decimal place so that deltaCn(1) is calculated by 1 − (0.0171654/0.0324374), which amount to 0.4708162 and then it is rounded to 0.4708

### E-value calculation

The purpose of “E-value calculation” is converting *R*_*new*_ into *R*_*new-E-value*_. For example, we explain how to calculate E-values of Comet. We built “Comet-E”, a modified version of Comet, to address the E-value correction. Comet-E has two more features than the original Comet. First, it can output the histogram of Xcorr values which was just an intermediate data structure used to calculate E-values in Comet. Second, it can take a histogram of Xcorr values as input and calculate E-values based on the histogram. Let His(*R*) denote a histogram for a result set *R*. We calculate His(*R*_*new*_) as follows: First, we run Comet-E to acquire histograms His(*R*_*del*_) and His(*R*_*ins*_). Then, His(*R*_*new*_) is calculated by “His(*R*_*old*_) − His(*R*_*del*_) + His(*R*_*ins*_)” where His(*R*_*old*_) was already produced earlier by Comet-E. Finally, His(*R*_*new*_) is given as input to Comet-E and it recalculates the E-value. Then, *R*_*new*_ is converted into *R*_*new-E-value*_. Detailed explanations are given in the following subsections i), ii), and iii). Subsection i) explains the E-value calculation by Comet and subsections ii) and iii) explain the two new features of Comet-E.

(i) **E-value calculation by Comet**

**Fig. 8:**
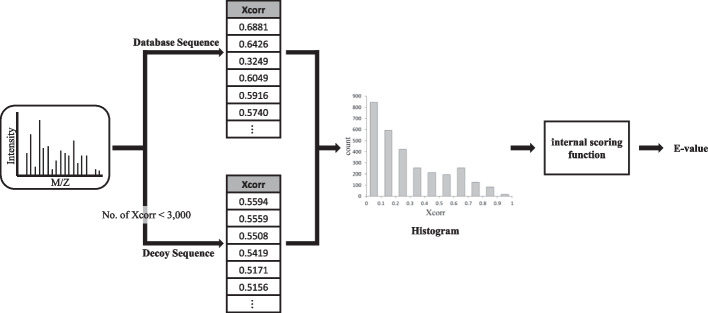
E-value calculation of Comet

Comet calculates the E-value for each spectrum based on Xcorr values for all candidate peptides (Fig. 8). Comet needs at least 3000 Xcorr values for each spectrum to calculate its E-value. Comet uses decoy peptides predefined in Comet if the number of Xcorr values is less than 3,000. Then, Comet calculates the histogram of the Xcorr values for each spectrum. The histogram is used to calculate the E-value of each spectrum by the internal scoring function of Comet.

(ii) **Comet-E (output)**

**Fig. 9:**
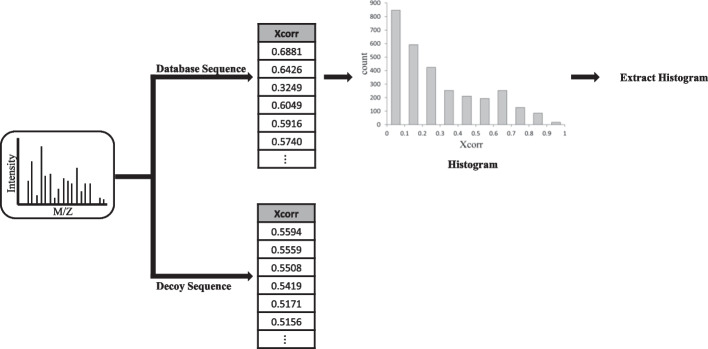
Histogram output by Comet-E

Comet-E can output the histogram of Xcorr values for each spectrum (Fig. 9). The histogram consists of Xcorr values for the sequences in the database only, excluding decoy sequences. Unlike Comet, Comet-E outputs the histogram table for every spectrum as a.txt file. Histograms are created with a bin width of 0.1, and has an average of 10 bin counts per spectrum. So, the histogram information (Xcorr counts for all bins) for each spectrum can be represented using only about 20 numbers.

(iii) **Comet-E (E-value recalculation)**

**Fig. 10:**
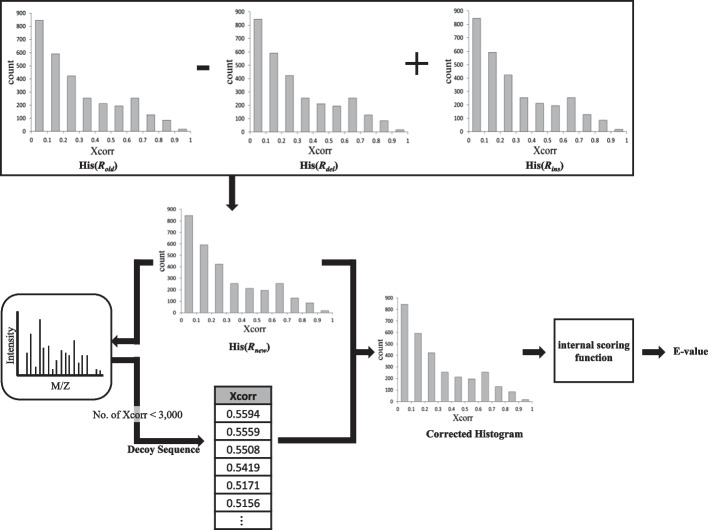
E-value recalculation by Comet-E

Given His(*R*_*new*_) as input, Comet-E can calculate the E-values of *R*_*new*_ (Fig. 10). Note that His(*R*_*new*_) is calculated by “His(*R*_*old*_) − His(*R*_*del*_) + His(*R*_*ins*_)”. This calculation is performed for each bin. If there is no output for a bin among histograms, its frequency is assigned to 0. Note that His(*R*_*old*_) was produced by Comet-E when *R*_*old*_ was generated and His(*R*_*del*_) and His(*R*_*ins*_) are produced by Comet-E when *R*_*del*_ and *R*_*ins*_ are generated, respectively. The time complexity of E-value calculation is O(|*S*|) because it is regardless of the size of database difference and only proportional to the number of spectra.

## Results

**Fig. 11:**
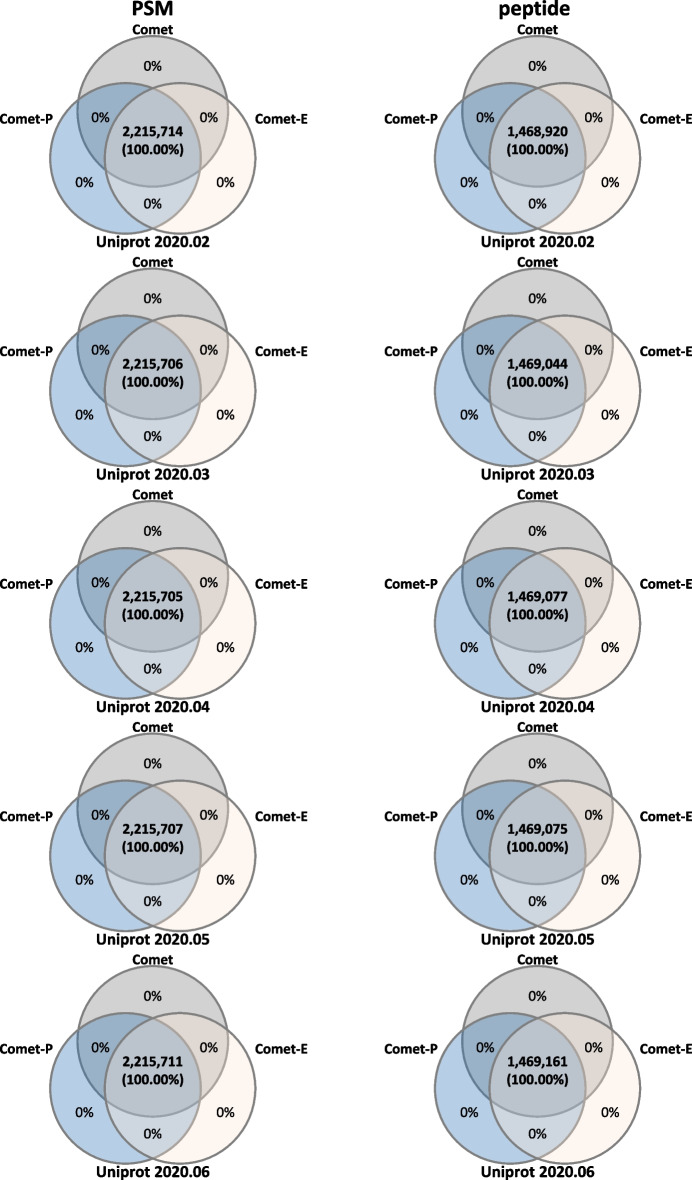
Consistency between Comet and Progressive Search results at the PSM and peptide level. For comparison, Progressive Search used the results analyzed using databases updated from Uniprot 2020.01 to Uniprot 2020.02, Uniprot 2020.03, Uniprot 2020.04, Uniprot 2020.05, and Uniprot 2020.06 versions. Comparisons were made for Comet, Comet-E and Comet-P for ntt1mc2

We measured and compared the running times of Comet, Comet-P (progressive Comet with PSM update only), and Comet-E (progressive Comet with both PSM and E-value update). The databases used were the SwissProt and TrEMBL human protein databases provided by UniProt. And tandem mass spectrometry (MS/MS) spectra for HEK293 cells [13] were used as an input, and the total number of spectra was 1,121,149. We compared them in different parameter settings: In subsection i), we show the results when the difference between *D*_*old*_ and *D*_*new*_ is fixed and the numbers of tryptic termini (ntt) and missed cleavages (mc) change. In subsection ii), we show the results when the difference between *D*_*old*_ and *D*_*new*_ changes and ntt and mc are fixed. The search results of Comet, Comet-P, and Comet-E remain consistent for both PSM and peptide levels (Fig. 11).

The entire experiments were carried out on a Linux PC with an Intel(R) Xeon(R) octa-core CPU E5-2609 v3 @ 1.90 GHz and 36 GB of RAM. The Linux version is Ubuntu 12.04.5 LTS and the compiler is GNU C compiler 6.5.0. All experiments were performed by a single thread.

(i) **Changing the numbers of tryptic termini and missed cleavages**

Table 2A shows the running time results when *D*_*old*_ and *D*_*new*_ are fixed to Uniprot 2020.01 and Uniprot 2020.02, respectively and ntt changes from 0 to 1 and mc changes from 0 to 2. Note that the difference between *D*_*old*_ (Uniprot 2020.01) and *D*_*new*_ (Uniprot 2020.02) is 0.16% (Fig. 4 #amino acid). Table 2A shows not only the overall running times of Comet, Comet-P, and Comet-E, but also breaks down the overall running times of Comet-E into the running times of individual modules (database separation, deletion, insertion, and E-value calculation). Note that the running time of Comet-P is the sum of the running times of all individual modules except the E-value calculation. For example, look at the leftmost column ntt2mc0. In this case, Comet, Comet-P, and Comet-E take 7846.9, 459.1, and 1900.1 s, respectively. The 459.1 s which is the running time of Comet-P is the sum of 3.7 s (database separation), 244.1 s (deletion), and 211.3 s (insertion). The 1900.1 s which is the running time of Comet-E is the sum of 459.1 s (Comet-P) and 1441.0 s (E-value calculation).

**Table 2:** Summary of the running times for various search parameter settings

	ntt2mc0	ntt2mc1	ntt2mc2	ntt1mc0	ntt1mc1	ntt1mc2
A (unit: second)						
1. Comet search	7846.9	7967.0	8320.1	15,084.1	25,650.1	34,287.5
2-a. Comet-P (without E-value)	459.1	447.9	442.0	528.0	575.3	635.9
2-b. Comet-E (with E-value)	1900.1	1709.3	1711.2	1911.1	2035.3	2075.7
2.1. Database separation	3.7	3.3	3.1	3.3	3.0	3.0
2.2. Deletion	244.1	228.2	223.1	261.5	280.2	344.3
2.2.1. Extract spectra	14.8	15.9	14.4	16.6	14.2	15.6
2.2.2. Comet-P/E search	72.5	57.9	58.4	78.5	91.8	158.7
2.2.3. Update results	156.9	154.3	150.3	166.4	174.2	170.0
2.3. Insertion	211.3	216.4	215.7	263.1	292.1	288.6
2.3.1. Comet-P/E search	168.9	168.6	162.9	192.6	216.1	213.7
2.3.2. Update results	42.4	47.8	52.8	70.5	76.0	74.9
2.4. E-value calculation	1441.0	1261.4	1269.3	1383.1	1459.9	1439.8
B						
The running time ratio						
Comet-P	5.85% (× 17.09)	5.62% (× 17.79)	5.31% (× 18.82)	3.50% (× 28.57)	2.24% (× 44.59)	1.85% (× 53.92)
Comet-E	24.21% (× 4.13)	21.45% (× 4.66)	20.57% (× 4.86)	12.67% (× 7.89)	7.93% (× 12.60)	6.05% (× 16.52)
Deletion	3.11%	2.86%	2.68%	1.73%	1.09%	1.00%
Insertion	2.69%	2.72%	2.59%	1.74%	1.14%	0.84%
E-value calculation	18.36%	15.83%	15.26%	9.17%	5.69%	4.20%
Database size ratio						
|*D*_*del*_ |/|*D*_*new*_|*	0.02% (8821 / 56,400,212)					
|*D*_*ins*_|/|*D*_*new*_|*	0.14% (76,988 / 56,400,212)					
Deleted PSMs ratio (|*S*_*del*_|/|S|)	0.02% (221 / 1,121,149)	0.02% (249 / 1,121,149)	0.02% (261 / 1,121,149)	0.02% (222 / 1,121,149)	0.02% (221 / 1,121,149)	0.02% (226 / 1,121,149)

(A) Running time comparison between Comet, Comet-P, and Comet-E in various search parameter settings. (B) Statistics of the running times. *The numerator and the denominator are the numbers of amino acids in target database only. If they are the numbers in both target and decoy databases, they are doubled and thus the ratio is still the same

Table 2B shows the statistics of the running time results in Table 2A. The running time ratio rows show the ratios of individual running times to the running time of Comet. Look at the leftmost column ntt2mc0 again. Since the running time of Comet-P is 459.1 s and that of the original Comet is 7846.9, the ratio is 459.1/7846.9 = 0.0585 = 5.85%. Since the ratio is 5.85%, Comet-P is 17.09 (= (1/5.85)*100) times faster than Comet which is shown just below 5.85% in the table. The speedup of Comet-P is between 17.09 (ntt2mc0) and 53.92 (ntt1mc2) and the speedup of Comet-E is between 4.13 (ntt2mc0) and 16.52 (ntt1mc2). Hence, the more nontryptic termini and missed cleavages there are, the bigger the speedup is.

Finally, it should be noted that the E-value calculation time does not change a lot as the nontryptic termini or missed cleavages change. It is between 1261.4 and 1459.9 s as shown in the last row of Table 2A. It may seem strange on a first look but it is reasonable because the time complexity of E-value calculation is just O(|*S*|) which means it is regardless of the size of database difference.

(ii) **Changing database update interval**

Table 3 shows the running times and their statistics of Comet, Comet-P, and Comet-E when ntt and mc are fixed to 1 and 2, respectively and the database update interval changes from 1 to 5 months. In this experiment, *D*_*old*_ is fixed to Uniprot 2020.01 and *D*_*new*_ changes appropriately from Uniprot 2020.02 to Uniprot 2020.06. The ratio |*D*_*del*_|/*|D*_*new*_| increases from 0.02% to 0.44% and the ratio |*D*_*ins*_|/*|D*_*new*_| also increases from 0.14% to 3.48% as the database update interval increases as shown in the last two rows in Table 3B. Recall that the time complexities of deletion and insertion are *O*(|*S*|∙|*D*_*del*_|) and *O*(|*S*|∙|*D*_*ins*_|), respectively. Thus, their running times are expected to increase as the database update interval increases. As expected, the measured running time of deletion (resp. insertion) increases from 344.3 to 598.3 s (resp. from 288.6 to 1443.9) as the database update interval increases as shown in Table 3A. When it comes to the E-value calculation, since its time complexity is O(|*S*|), its running time is regardless of the database update interval. It is between 1394.5 and 1496.7 s. Conclusively, the speedup of Comet-P is between 53.92 (1 month) and 17.39 (5 months) and the speedup of Comet-E is between 16.52 (1 month) and 10.23 (5 months) as shown in Table 3B.

**Table 3:** Summary of the running times for several database update intervals

	1 month	2 months	3 months	4 months	5 months
A (unit: second)					
1. Comet search	34,287.5	35,073.6	35,052.1	34,840.2	35,559.4
2-a. Comet-P (without E-value)	635.9	1282.6	1439.5	1532.8	2045.3
2-b. Comet-E (with E-value)	2075.7	2779.3	2834.0	2960.5	3476.1
2.1. Database separation	3.0	3.0	3.0	3.2	3.1
2.2. Deletion	344.3	410.0	459.0	498.8	598.3
2.2.1. Extract spectra	15.6	15.8	15.0	16.3	15.7
2.2.2. Comet-P/E search	158.7	184.9	199.5	216.2	251.7
2.2.3. Update results	170.0	209.3	244.5	266.3	330.8
2.3. Insertion	288.6	869.6	977.5	1030.8	1443.9
2.3.1. Comet-P/E search	213.7	787.8	893.9	946.9	1354.8
2.3.2. Update results	74.9	81.8	83.6	83.9	89.1
2.4. E-value calculation	1439.8	1496.7	1394.5	1427.8	1430.8
B					
The running time ratio					
Comet-P	1.85% (× 53.92)	3.66% (× 27.35)	4.11% (× 24.35)	4.40% (× 22.73)	5.75% (× 17.39)
Comet-E	6.05% (× 16.52)	7.92% (× 12.62)	8.09% (× 12.37)	8.50% (× 11.77)	9.78% (× 10.23)
Deletion	1.00%	1.17%	1.31%	1.43%	1.68%
Insertion	0.84%	2.48%	2.79%	2.96%	4.06%
E-value calculation	4.20%	4.27%	3.98%	4.10%	4.02%
Database size ratio					
|*D*_*del*_ |/|*D*_*new*_|*	0.02% (8821 / 56,400,212)	0.11% (64,323 / 57,348,066)	0.21% (118,748 / 57,471,311)	0.26% (149,505 / 57,516,232)	0.44% (252,754 / 58,101,266)
|*D*_*ins*_|/|*D*_*new*_|*	0.14% (76,988 / 56,400,212)	1.88% (1,080,344 / 57,348,066)	2.19% (1,258,014 / 57,471,311)	2.32% (1,333,692 / 57,516,232)	3.48% (2,021,975 / 58,101,266)
Deleted PSMs ratio (|*S*_*del*_|/|S|)	0.02% (226 / 1,121,149)	0.10% (1121 / 1,121,149)	0.15% (1717 / 1,121,149)	0.21% (2410 / 1,121,149)	0.31% (3426 / 1,121,149)

(A) Running time Comparison between Comet, Comet-P, and Comet-E with several database update intervals. (B) Statistics of the running times. *The numerator and the denominator are the numbers of amino acids in target database only. If they are the numbers in both target and decoy databases, they are doubled and thus the ratio is still the same

## Conclusions

Progressive search is a novel approach to efficiently obtain analysis results for updated database in tandem mass spectrometry. Its running time is *O*(|*S|*|*ΔD|*) on average and thus it is up to 53.9 times faster than the normal search from scratch for PSM update only (including the update of PSM scores such as Xcorr and DeltaCn) and up to 16.5 times faster for both PSM and E-value update for the intervals up to 5 months. We also discovered our Progressive search is effective even for longer intervals. Comet-P and Comet-E are 2.5 and 4 times faster than normal search, respectively, even with the interval of 34 months (July 2019 and May 2022 databases) (data not shown). The PSMs and E-values achieved by progressive search are the same as those achieved by the normal search from scratch. In addition, we verified that repeated use of progressive search does not increase the differences in deltaCn values due to rounding. We compared the results from searches for 3-month intervals (between Jan. 2020 and Apr. 2020) with results from 3 repeated searches for 1-month interval (between Jan. 2020 and Feb. 2020, between Feb. 2020 and Mar. 2020, and between Mar. 2020 and Apr. 2020). The deltaCn values were the same in both results although progressive search was used multiple times. This study demonstrates the applicability of Progressive search for efficient tandem mass spectrometry database search. Use of this approach can be extended to a variety of public search tools, including Comet.

### Availability and requirements

Project name: progressive search.

Project home page: https://isa.hanyang.ac.kr/ProgSearch.html

Operating system(s): Linux.

Programming language: Java, C +  + 

Other requirements: JDK 1.8 or higher.

License: Apache License V2.0

Any restrictions to use by non-academics: as stipulated by Apache License V2.0

## Data Availability

Experiments were carried out with the August 2019 version of Comet and can be obtained through Comet website: http://comet-ms.sourceforge.net. The database used in this current study are publicly available in the UniProt website: https://www.uniprot.org. The databases used were the SwissProt and TrEMBL human protein databases provided by UniProt. We measured the performance of Progressive Search using tandem mass spectrometry (MS/MS) spectra for HEK293 cells [13]. The HEK293 24-fraction MS/MS dataset was used in the experiment, and the total number of spectra was 1,121,149.

## Abbreviations

*S*: Set of spectra
*D*: Set of peptides in a database
*D*_*new*_: New database
*D*_*old*_: Old database
*D*_*srd*_: Database which contains the proteins shared by both *D*_*old*_ and *D*_*new*_
*D*_*del*_: Database which contains the proteins stored in only *D*_*new*_
*D*_*ins*_: Database which contains the proteins stored in only *D*_*new*_
*R*_*new*_: PSM results for *D*_*new*_
*R*_*old*_: PSM results for *D*_*old*_
*R*_*srd*_: PSM results for *D*_*srd*_
*R*_*del*_: PSM results for *D*_*del*_
*R*_*ins*_: PSM results for *D*_*ins*_
